# Supplementary material for: Molecular surveillance over 14 years confirms reduction of Plasmodium vivax and falciparum transmission after implementation of Artemisinin-based combination therapy in Papua, Indonesia
Source: PLoS Negl Trop Dis. 2020 May 7;14(5):e0008295. doi: 10.1371/journal.pntd.0008295 (PMC7237043; doi:10.1371/journal.pntd.0008295)
Supplement: S1 Table — (DOC) [file pntd.0008295.s001.doc]

**S1 Table. Demographic data by temporal period for *P. falciparum* and *P. vivax* isolates included in the study versus all cases screened.**

| ***P. vivax*** | | **2004-2006** | | **2006-2009** | | **2009-2012** | | **2012-2015** | | **2015-2017** | |
| --- | --- | --- | --- | --- | --- | --- | --- | --- | --- | --- | --- |
| **(N=1,197)** | | **All^12,12,21^** | **Genotyped^13,13,22^** | **All^9,9,10^,** | **Genotyped^7,7,8^** | **All^7,7,7^** | **Genotyped^7,7,7^** | **All^2,2,3^** | **Genotyped^2,2,3^** | **All^7,8,7^** | **Genotyped^7,8,7^** |
| **Age Group,**  **n (%)** | **Children <15 years** | 267 (47) | 36 (32) | 39 (19) | 31 (23) | 30 (25) | 28 (26) | 29 (19) | 24 (17) | 27 (25) | 20 (23) |
|  | **Adults ≥ 15 years** | 303 (53) | 78 (68) | 171 (81) | 105 (77) | 90 (75) | 79 (74) | 124 (81) | 120 (83) | 80 (75) | 66 (77) |
| **Males n (%)** | | 314 (54) | 142 (56) | 85 (39) | 118 (44) | 65 (51) | 104 (48) | 66 (43) | 130 (40) | 52 (46) | 112 (51) |
| **Parasitaemia Parasites/µL** | | 1,902 | 2,496 | 9,230 | 9,454 | 15,568 | 16,784 | 15,609 | 15,634 | 10,143 | 10,541 |
| GM (95%CI) | | (1,652-2,190) | (1,825-3,414) | (8,313-10247) | (8,290-10,782) | (13,189-18,377) | (14,113-19,959) | (13,187-18,475) | (13,153-18,584) | (8,926-11,525) | (9,116-12,189) |
| **Total n (%)** | | 582 | 119 (20) | 219 | 143 (65) | 127 | 114 (90) | 155 | 146 (94) | 114 | 93 (82) |
| ***P. falciparum*** | | **2004-2006** | | **2006-2009** | | **2009-2012** | | **2012-2015** | | **2015-2017** | |
| **(N=1,566)** | | **All^10,8,44^** | **Genotyped^5,5,2^** | **All^10,9,4^** | **Genotyped^6,5,2^** | **All^6,6,3^** | **Genotyped^5,5,3^** | **All^0,0,5^** | **Genotyped^0,0,4^** | **All^11,11,13^** | **Genotyped^11,11,13^** |
| **Age Group**  **n (%)** | **Children <15 years** | 364 (40) | 20 (15) | 9 (5) | 5 (4) | 20 (18) | 17 (18) | 22 (12) | 21 (12) | 34 (26) | 27 (23) |
|  | **Adults ≥ 15 years** | 551 (60) | 110 (85) | 180 (95) | 117 (96) | 94 (82) | 80 (82) | 157 (88) | 155 (88) | 98 (74) | 87 (76) |
| **Males n (%)** | | 554 (60) | 80 (59) | 88 (44) | 60 (47) | 57 (48) | 49 (48) | 70 (39) | 69 (39) | 81 (57) | 71 (57) |
| **Parasitaemia Parasites/µL** | | 4,025 | 10,423 | 14,755 | 14,254 | 19,996 | 20,327 | 19,547 | 19,327 | 18,010 | 18,981 |
| GM (95%CI) | | (3,582-4,523) | (8,750-12,416) | (12,628-17,240) | (12,044-16,870) | (16,778-23,830) | (16,730-24,696) | (16,979-22,502) | (16,813-22,216) | (15,273-21,238) | (15,856-22,722) |
| **Total n (%)** | | 925 | 135 (15) | 199 | 128 (64) | 120 | 102 (85) | 179 | 176 (98) | 143 | 125 (83) |

GM: Geometric mean; 95%CI: 95% Confidence interval; # Superscript indicate number of missing data for Age, Sex and Parasitaemia
