# Supplementary material for: Molecular surveillance over 14 years confirms reduction of Plasmodium vivax and falciparum transmission after implementation of Artemisinin-based combination therapy in Papua, Indonesia
Source: PLoS Negl Trop Dis. 2020 May 7;14(5):e0008295. doi: 10.1371/journal.pntd.0008295 (PMC7237043; doi:10.1371/journal.pntd.0008295)
Supplement: S2 Table — (DOC) [file pntd.0008295.s002.doc]

***S2 Table.*** *Marker diversity and genotyping success rate in P. vivax and P. falciparum*.

| **Species** | **Markers** | **2004 - 2006** | **Success, N (%)** | **2006 - 2009** | **Success, N (%)** | **2009 - 2012** | **Success, N (%)** | **2012 - 2015** | **Success, N (%)** | **2015 - 2017** | **Success, N (%)** |
| --- | --- | --- | --- | --- | --- | --- | --- | --- | --- | --- | --- |
|  |  | ***H_E_*** |  | ***H_E_*** |  | ***H_E_*** |  | ***H_E_*** |  | ***H_E_*** |  |
| ***P. vivax*** | *MS1* | 0.779 | 111 (90) | 0.781 | 143 (99) | 0.787 | 111 (195) | 0.774 | 138 (93) | 0.795 | 91 (96) |
|  | *MS10* | 0.898 | 118 (96) | 0.905 | 142 (98) | 0.895 | 112 (96) | 0.892 | 141 (95) | 0.902 | 84 (88) |
|  | *MS12* | 0.768 | 117 (95) | 0.786 | 141 (98) | 0.760 | 106 (91) | 0.772 | 137 (92) | 0.753 | 92 (97) |
|  | *MS16* | 0.923 | 107 (87) | 0.936 | 138 (96) | 0.941 | 96 (82) | 0.917 | 122 (82) | 0.959 | 89 (94) |
|  | *MS20* | 0.922 | 112 (91) | 0.910 | 140 (97) | 0.907 | 102 (87) | 0.903 | 135 (91) | 0.898 | 92 (97) |
|  | *MS5* | 0.869 | 120 (98) | 0.850 | 141 (98) | 0.862 | 103 (88) | 0.873 | 133 (89) | 0.818 | 91 (96) |
|  | *msp1f3* | 0.864 | 117 (95) | 0.834 | 140 (97) | 0.780 | 104 (89) | 0.798 | 136 (91) | 0.856 | 88 (93) |
|  | *pv3.27* | 0.89 | 113 (92) | 0.86 | 137 (95) | 0.883 | 105 (90) | 0.900 | 133 (89) | 0.900 | 87 (92) |
|  | ***Total*** | 0.864 | 123 | 0.858 | 144 | 0.852 | 117 | 0.854 | 149 | 0.86 | 95 |
| ***P. falciparum*** | *ARAII* | 0.744 | 135 (100) | 0.823 | 129 (99) | 0.797 | 101 (99) | 0.791 | 176 (99) | 0.807 | 125 (98) |
|  | *PfPK2* | 0.776 | 130 (96) | 0.770 | 129 (99) | 0.721 | 101 (99) | 0.644 | 175 (99) | 0.511 | 125 (98) |
|  | *Polyalpha* | 0.761 | 134 (99) | 0.749 | 126 (97) | 0.751 | 100 (98) | 0.623 | 176 (99) | 0.516 | 125 (98) |
|  | *TA1* | 0.633 | 135 (100) | 0.714 | 129 (99) | 0.681 | 101 (99) | 0.615 | 176 (99) | 0.593 | 122 (96) |
|  | *TA109* | 0.113 | 134 (99) | 0.016 | 128 (98) | 0 | 101 (99) | 0 | 176 (99) | 0.337 | 124 (98) |
|  | *TA42* | 0.313 | 132 (98) | 0.431 | 129 (99) | 0.483 | 101 (99) | 0.189 | 175 (99) | 0.430 | 121 (95) |
|  | *TA60* | 0.623 | 134 (99) | 0.640 | 129 (99) | 0.680 | 101 (99) | 0.687 | 175 (99) | 0.723 | 125 (98) |
|  | *TA81* | 0.715 | 129 (96) | 0.794 | 128 (98) | 0.815 | 100 (98) | 0.781 | 176 (99) | 0.787 | 125 (98) |
|  | *TA87* | 0.671 | 129 (96) | 0.710 | 128 (98) | 0.679 | 100 (98) | 0.578 | 172 (97) | 0.710 | 125 (98) |
|  | ***Total*** | 0.594 | 135 | 0.628 | 130 | 0.623 | 102 | 0.545 | 177 | 0.602 | 127 |
