# Supplementary material for: Molecular surveillance over 14 years confirms reduction of Plasmodium vivax and falciparum transmission after implementation of Artemisinin-based combination therapy in Papua, Indonesia
Source: PLoS Negl Trop Dis. 2020 May 7;14(5):e0008295. doi: 10.1371/journal.pntd.0008295 (PMC7237043; doi:10.1371/journal.pntd.0008295)
Supplement: S3 Table — GM: Geometric mean; 95%CI: 95% Confidence interval. (DOC) [file pntd.0008295.s003.doc]

**S3 Table.** Demographic data by temporal period.

| **Periods** | **Age n (%)** | | **Male**  **n (%)** | **Parasitaemia (p/µL)** | **Total** |
| --- | --- | --- | --- | --- | --- |
|  | **<15 years** | **> 15 years** |  | **Geometric Mean (CI95%)** |  |
| ***P. vivax*** | | | | | |
| 2004–2006 | 31 (36/115) | 69 (79/115) | 53 (63/115) | 15,506 (12,839-18,728) | 119 |
| 2006-2009 | 23 (31/137) | 77 (106/137) | 41 (58/137) | 9,445 (8,257-10,805) | 143 |
| 2009-2012 | 26 (28/107) | 74 (79/107) | 48 (55/107) | 16,697 (14,165-19,682) | 114 |
| 2012–2015 | 17 (24/144) | 83 (120/144) | 42 (61/144) | 14,941 (12,703-17,573) | 146 |
| 2015–2017 | 23 (20/86) | 77 (66/86) | 44 (41/85) | 10,176 (8,790-11,780) | 93 |
| **Total** | 24 (139/589) | 76 (450/589) | 45 (278/588) | 9309 (8394-10323) | 615 |
| ***P. falciparum*** | | | | | |
| 2004–2006 | 16 (20/129) | 84 (109/129) | 59 (79/130) | 10,423 (8,750-12,416) | 135 |
| 2006-2009 | 4 (5/122) | 96 (117/122) | 47 (60/123) | 14,254 (12,044-16,870) | 128 |
| 2009-2012 | 18 (17/97) | 82 (80/97) | 48 (49/97) | 20,327 (16,730-24,696) | 102 |
| 2012–2015 | 12 (21/176) | 88 (155/176) | 39 (69/176) | 19327 (16,813-22,216) | 176 |
| 2015–2017 | 24 (27/114) | 76 (87/114) | 57 (71/114) | 18,981 (15,856-22,722) | 125 |
| **Total** | 14 (90/638) | 86 (548/638) | 49 (328/640) | 16097 (14,893-17,397) | 666 |

GM: Geometric mean; 95%CI: 95% Confidence interval
