# Supplementary material for: Molecular surveillance over 14 years confirms reduction of Plasmodium vivax and falciparum transmission after implementation of Artemisinin-based combination therapy in Papua, Indonesia
Source: PLoS Negl Trop Dis. 2020 May 7;14(5):e0008295. doi: 10.1371/journal.pntd.0008295 (PMC7237043; doi:10.1371/journal.pntd.0008295)
Supplement: S4 Table — (DOC) [file pntd.0008295.s004.doc]

***S4 Table.*** *Ancestry of P. falciparum isolates assuming 2 and 4 sub-populations*.

| **Period** | **K1, N (%)** | **K2, N (%)** | **K3, N (%)** | **K4, N (%)** | **Admixture, N** | **Total** |
| --- | --- | --- | --- | --- | --- | --- |
| ***delta K* = 2** | | | | | | |
| **2004–2006** | 66 (69) | 29 (31) | - | - | 40 | 135 |
| **2006-2009** | 52 (59) | 36 (41) | - | - | 40 | 128 |
| **2009-2012** | 42 (62) | 26 (38) | - | - | 34 | 102 |
| **2012–2015** | 104 (86) | 17 (14) | - | - | 55 | 176 |
| **2015–2017** | 59 (63) | 35 (37) | - | - | 31 | 125 |
| **Total** | 323 | 143 | - | - | 200 | 666 |
| ***delta K* = 4** | | | | | | |
| **2004–2006** | 29 (48) | 0 (0) | 28 (46) | 4 (7) | 74 | 135 |
| **2006-2009** | 28 (40) | 14 (20) | 25 (36) | 3 (4) | 58 | 128 |
| **2009-2012** | 9 (20) | 15 (34) | 15 (34) | 5 (11) | 58 | 102 |
| **2012–2015** | 34 (45) | 0 (0) | 15 (20) | 27 (36) | 100 | 176 |
| **2015–2017** | 6 (7) | 25 (29) | 13 (15) | 42 (49) | 39 | 125 |
| **Total** | 106 | 54 | 96 | 81 | 329 | 666 |
