# Supplementary figures and images for: Molecular surveillance over 14 years confirms reduction of Plasmodium vivax and falciparum transmission after implementation of Artemisinin-based combination therapy in Papua, Indonesia
Source: PLoS Negl Trop Dis. 2020 May 7;14(5):e0008295. doi: 10.1371/journal.pntd.0008295 (PMC7237043; doi:10.1371/journal.pntd.0008295)

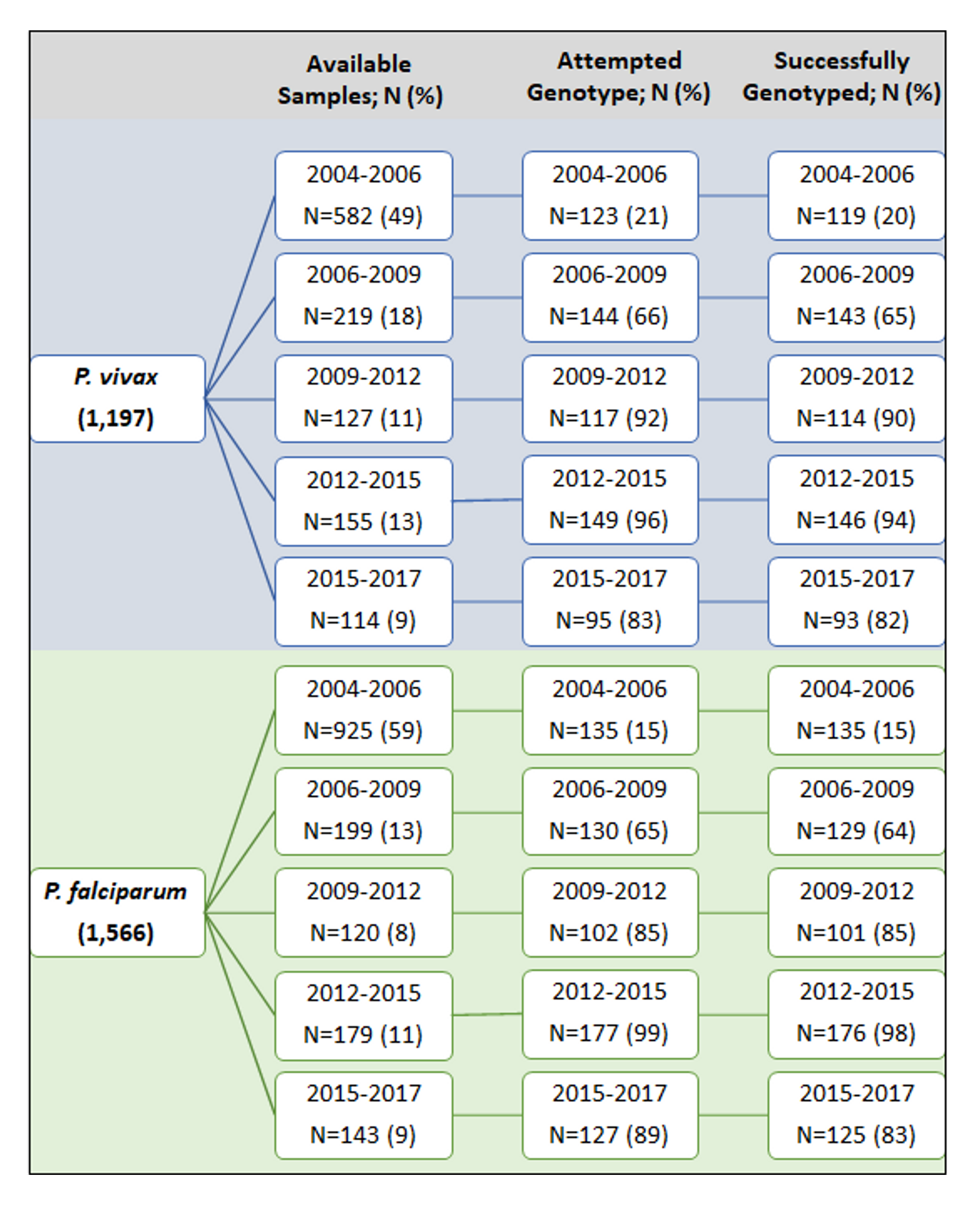

Supplement: S1 Fig — (TIFF) [file pntd.0008295.s005.tiff]

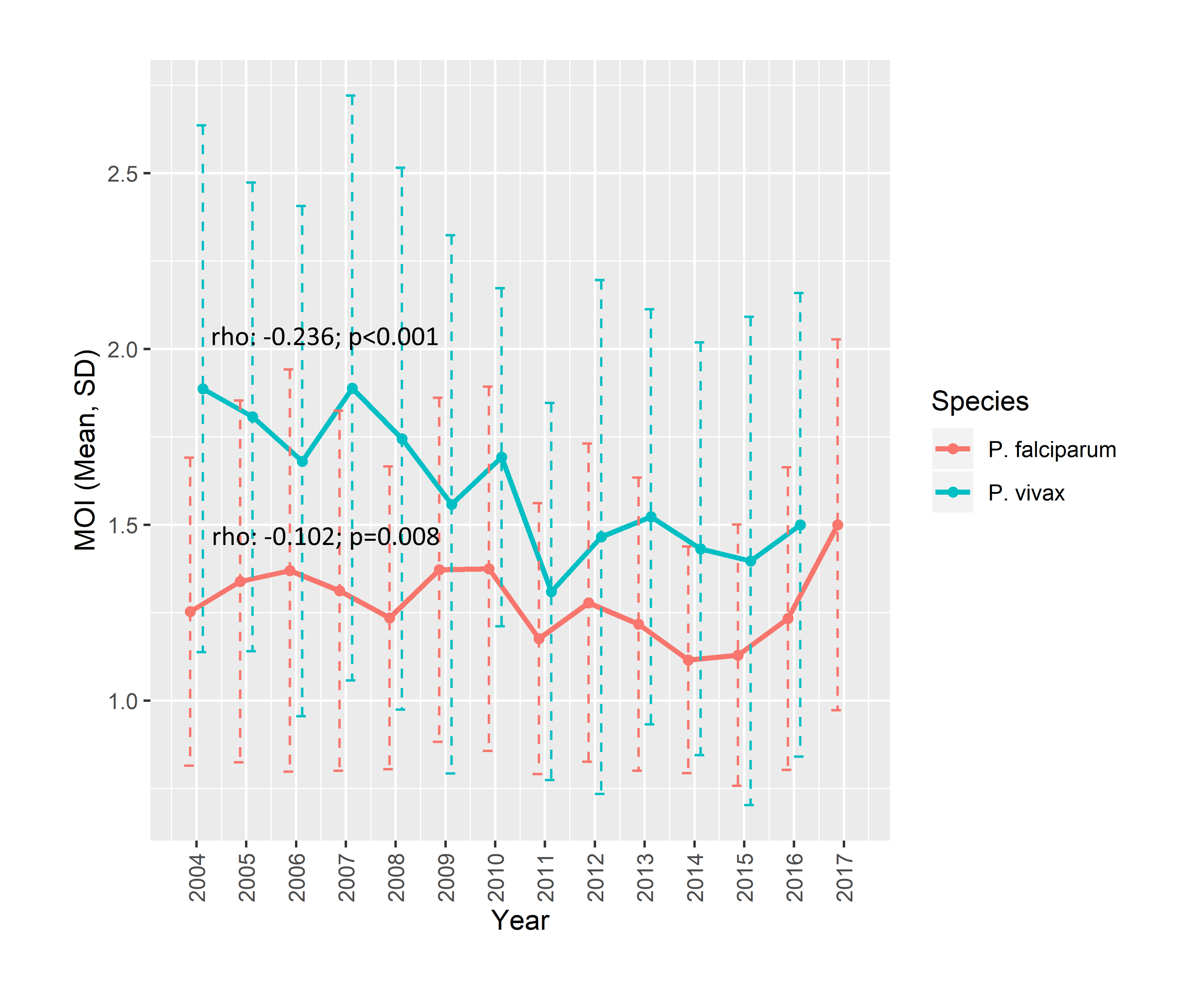

Supplement: S2 Fig — (TIFF) [file pntd.0008295.s006.tiff]

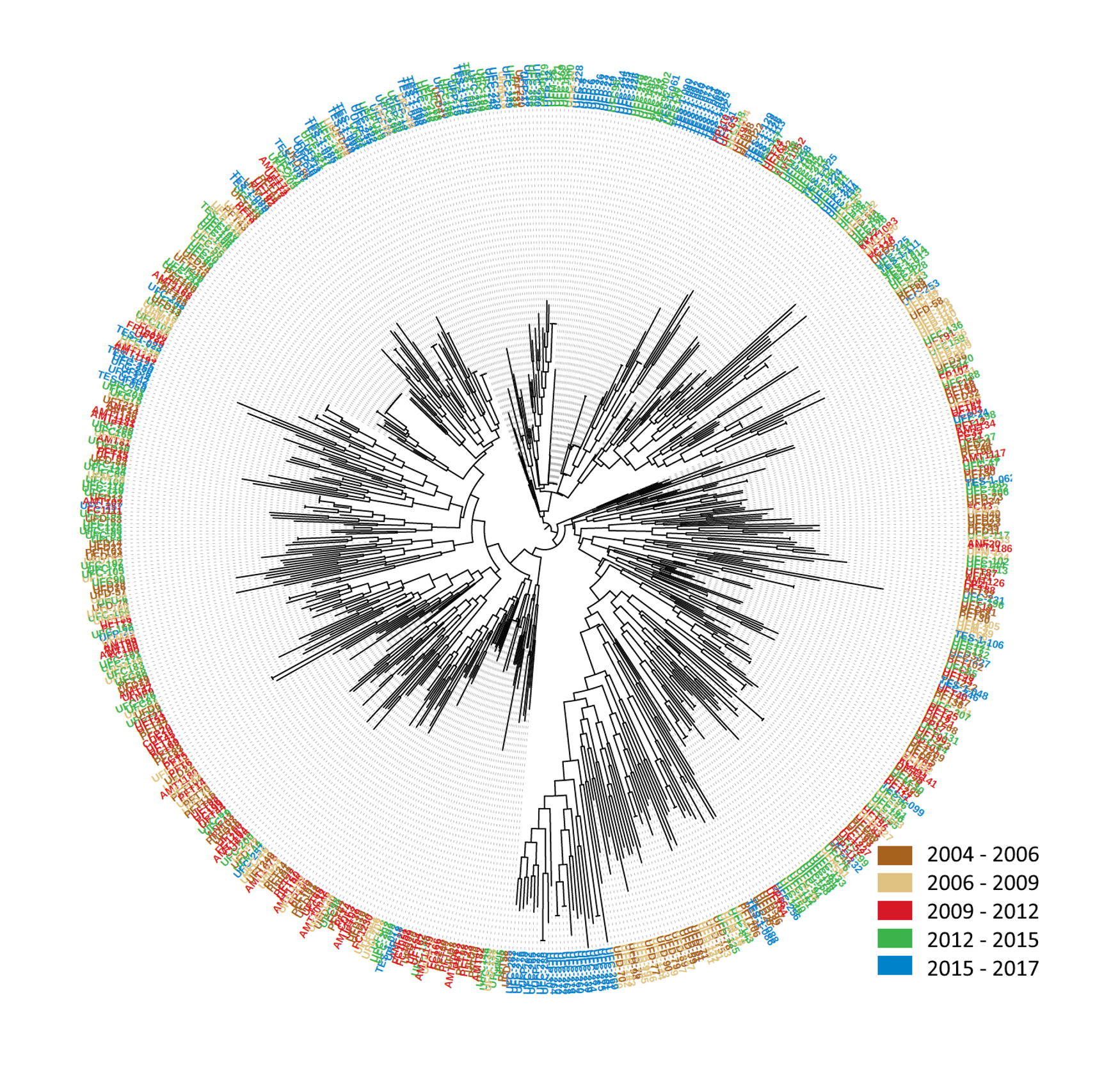

Supplement: S3 Fig — (TIFF) [file pntd.0008295.s007.tiff]

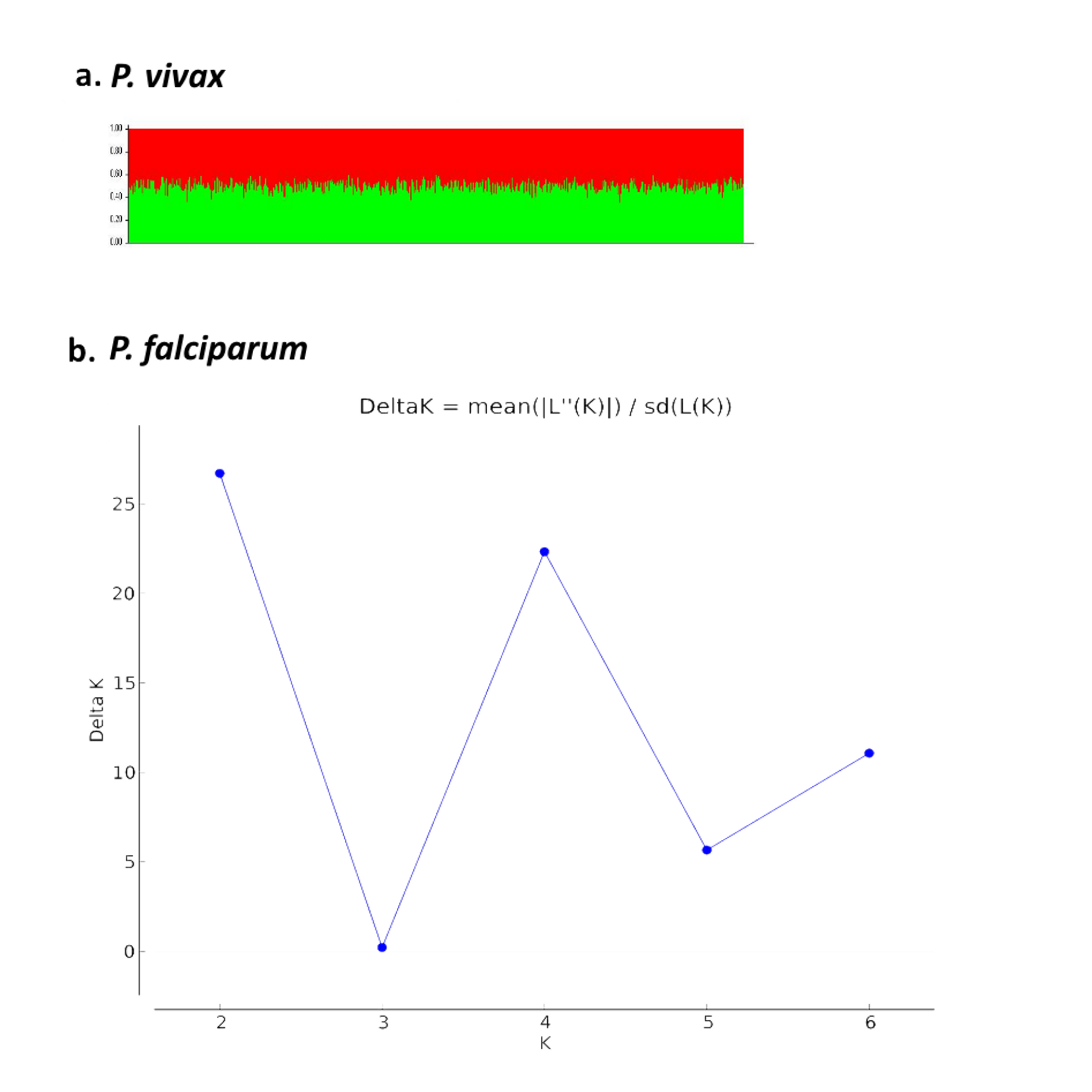

Supplement: S4 Fig — Panel a) provides a STRUCTURE bar plot constructed from the P. vivax data at K = 2, illustrating a lack of notable sub-structure. Panel b) provides a scatter plot of K against Delta K for the P. falciparum data, illustrating peaks at K = 2 and K = 4. (TIFF) [file pntd.0008295.s008.tiff]

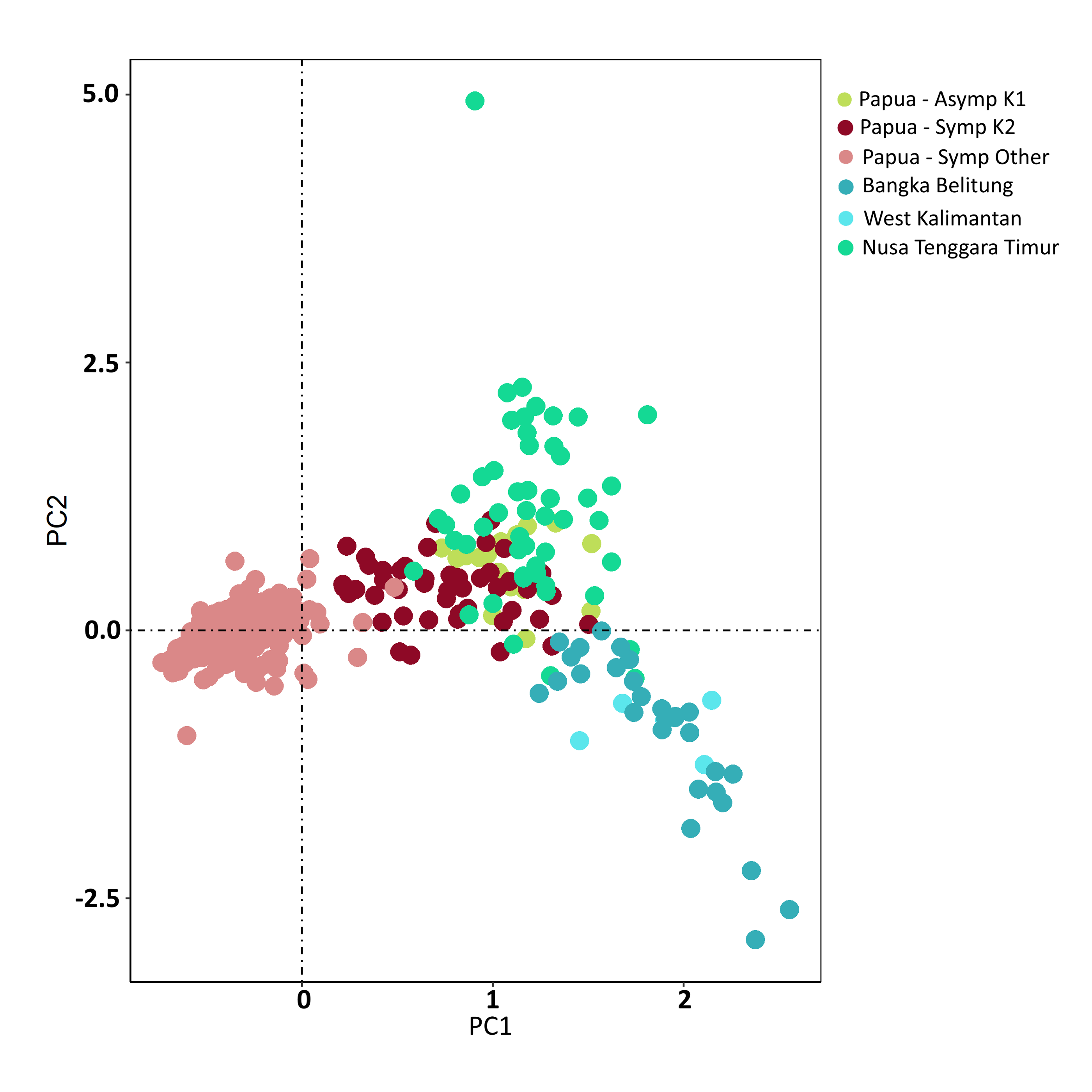

Supplement: S5 Fig — Higher genetic relatedness was observed between the putatively imported Papuan asymptomatic K1 subpopulation (Papua Asymp K1, green circles) [15], the Papuan symptomatic K2 subpopulation (Papua Symp K2, red circles), and the infections from Nusa Tenggara Timur (aquamarine circles)[16] than the other Papuan symptomatic infections from the current study (Papua Symp Other, pink circles). (TIFF) [file pntd.0008295.s009.tiff]

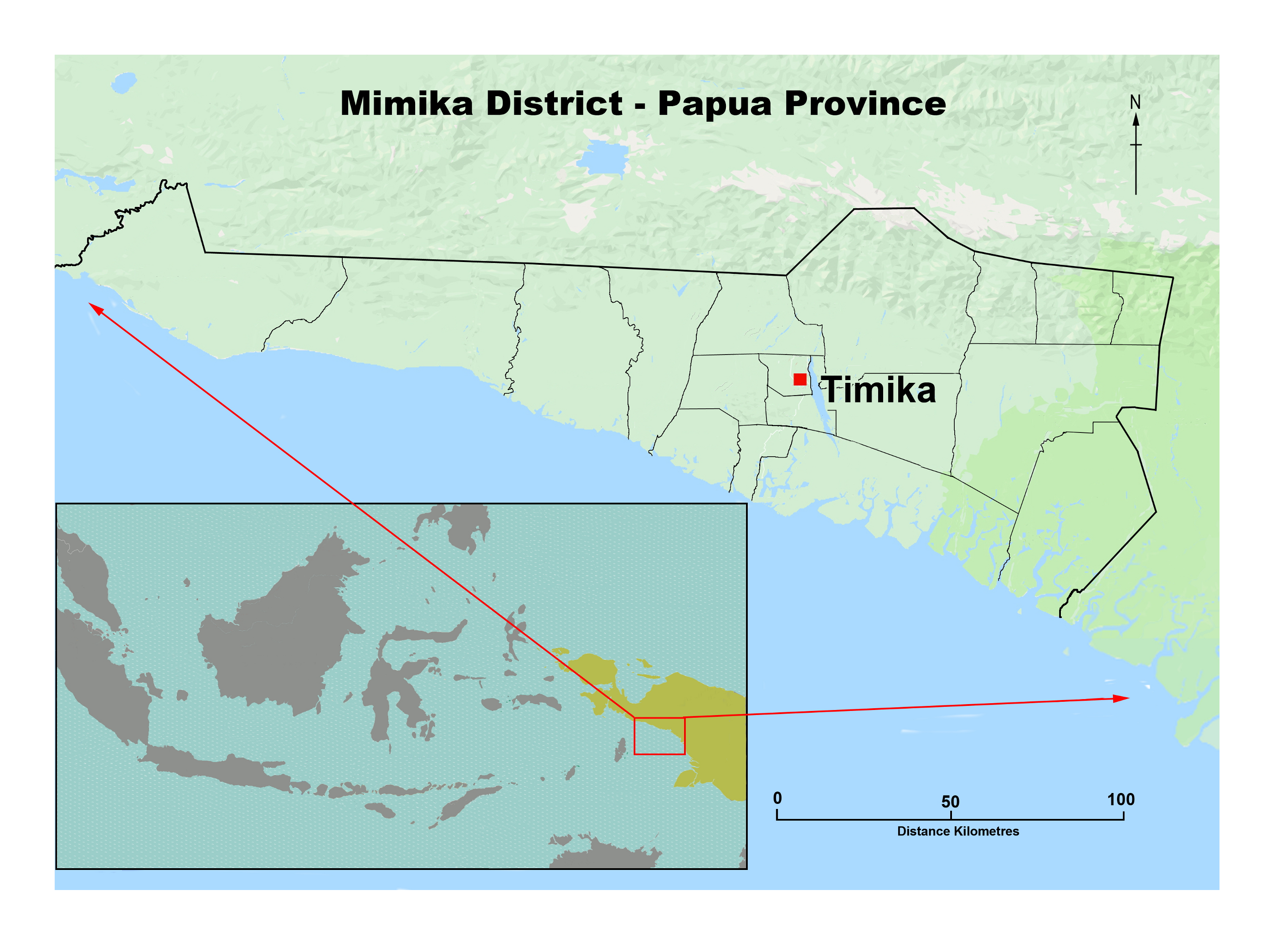

Supplement: S6 Fig — Map adapted from Kenangalem et al. 2019 illustrating the location of Mimika District within Papua Province, Indonesia. (TIFF) [file pntd.0008295.s010.tiff]
