# Supplementary material for: Molecular surveillance over 14 years confirms reduction of Plasmodium vivax and falciparum transmission after implementation of Artemisinin-based combination therapy in Papua, Indonesia
Source: PLoS Negl Trop Dis. 2020 May 7;14(5):e0008295. doi: 10.1371/journal.pntd.0008295 (PMC7237043; doi:10.1371/journal.pntd.0008295)
Supplement: Attachment — Submitted filename: PrePost_ACT_Revision_Cover_Letter_23012020.pdf [file pntd.0008295.s011.pdf]

menzies
school of health research

PO Box 41096, Casuarina NT 0811, Australia
John Mathews Building (Bldg 58),
Royal Darwin Hospital Campus, Rocklands Dve, Casuarina NT 0810
Ph: 08 8922 8196 | Fax: 08 8927 5187 | Web: www.menzies.edu.au
ABN: 70 413 542 847

discovery for a healthy tomorrow

23rd January 2020

Academic Editors
PLoS NTD

Dear Dr Deye and Dr Dutra,

Manuscript reference number: **PNTD-D-19-01519**

"Longitudinal molecular surveillance confirms interruption of *P. vivax* and *P. falciparum* transmission following implementation of a universal policy of Artemisinin-based Combination Therapy in Papua, Indonesia"

We thank you for the opportunity to revise and resubmit our manuscript. We also thank the peer reviewer whose positive and constructive comments have helped us to improve the manuscript. As detailed below, we have provided revisions with consideration of the reviewers' suggestions.

We look forward to receiving further feedback from you once additional reviews are available.

Yours sincerely,

Dr Sarah Auburn

Senior Research Fellow, PhD

Reviewer comments RE Methods

1. ***Methods are mostly adequate for the aims of the study. I note, however, that a population bottleneck is mentioned (e.g., Author Summary and Discussion, line 292) but no formal test for bottleneck was applied to the samples.***

We have acknowledged the reviewer's suggestion, and now include tests of excess heterozygosity using three different models to search for evidence of bottlenecking in the different study periods in both species. Details on the methods are provided in lines 444-449 as follows:

"We used the heterozygosity excess test implemented in the Bottleneck software version 1.2.02 to determine if population size had recently suffered a reduction. We considered the study periods as populations, and tested three different models; an Infinite Alleles Model (IAM), a strict Stepwise Mutation Model (SMM), and a Two Phase Mutational (TPM) model of 70% stepwise and 30% non-stepwise mutations, running 10,000 iterations for each model [41]."

The results of the bottleneck tests are presented in lines 172-178 and a new table (Table 3) and discussed in lines 287-306. In summary, we found evidence of bottlenecking when applying the Infinite Allele Model to test for evidence of excess heterozygosity. However, the Two-Phase Mutational model did not find evidence of bottlenecking in any study period in either species, and we have some concerns about the sensitivity of this approach to detect subtle changes.

2. ***A LD test was correctly used but described in a rather weird way. In fact, the standardised index of association implemented in LIAN software does not "compare the observed variance in the numbers of alleles shared between parasites with that expected when parasites share no alleles at different loci", as stated in lines 393-395. In fact, the index compares the observed variance of the number of alleles at which each pair of haplotypes differ in the population (i.e., the allele mismatch distribution) with the variance expected under random association of alleles. This is very important, because parasites may share identical alleles in a panmictic population -- therefore, the null hypothesis does not imply "no alleles shared"!***

We apologise for any confusion. The analysis of LD was conducted using the approach described by the review (using LIAN software). The text (lines 437-440) has now been revised to more accurately describe this method as follows:

"LD, which is the non-random association of alleles at different loci, was measured using the standardised index of association (I_A^S). I_A^S compares the observed variance of the number of mismatched loci between haplotypes to the expected variance if the loci were randomly associated."

Reviewer comments RE Results

Overall, results are well presented in the main text. Tables are clear and serve the purpose of presenting the main findings.

Figure 1 is ok but may be misleading; a pie chart would possibly be more adequate for the purpose of showing the, among multiple-clone infections, the proportion of those with 1 or more alleles found at a single or a few loci tends to increase with time. The bar chart may give the impression of an increasing overall prevalence of multiple-clone infections, which is clearly not true.

Figure 2 is not very effective in presenting the results. A new figure inspired in Figures 1 and 3 of reference 9, for example, can be a better option. The point here is to show that particular haplotypes shared by two or more isolates persist over time.

We thank the reviewer for their advice. Figures 1 and 2 have been revised in accordance.

<u>Reviewer comments RE Conclusions</u>

Most conclusions are supported by the data, but a few important exceptions must be mentioned. First, the title: "Longitudinal molecular surveillance confirms interruption ...". Overall, the article is nicely written, the sample size is large enough for the proposed analysis, results are clearly presented... but the title is completely misleading. Data is this paper does not "confirm" that malaria transmission has been interrupted in Papua! Such a "confirmation" would require evidence that all remaining cases are imported, not locally transmitted, and no data support this! I would suggest that the data are consistent with a decrease in malaria transmission (mostly that of P. falciparum) following the implementation of the universal ACT policy. That is all.

The title was intended to imply <u>partial interruption</u> to transmission, not complete interruption, given that there is clearly still transmission in the study region. We appreciate that this may not have been clear and have revised the title to “Longitudinal molecular surveillance confirms reduction of *P. vivax* and *P. falciparum* transmission following implementation of a universal policy of Artemisinin-based Combination Therapy in Papua, Indonesia” to avoid confusion to any other readers.

What is consistent with transmission decline? Essentially, the decrease in the prevalence of multiple-clone infections and in average multiplicity of infection over time.

Is increased LD consistent with decreased transmission. Not necessarily. LD is a consequence of reduced recombination and, of course, any factor reducing recombination will affect LD estimates. For example, malaria incidence may increase due to a clonal outbreak -- with increased transmission and increased LD. Therefore, it is important to document changes in LD over time, but they do not "confirm" that malaria transmission has declined.

As stated in the text (lines 334-336), three genetic indices correlated with declining transmission in one or both species:

menzies
school of health research

PO Box 41096, Casuarina NT 0811, Australia
John Mathews Building (Bldg 58),
Royal Darwin Hospital Campus, Rocklands Dve, Casuarina NT 0810
Ph: 08 8922 8196 | Fax: 08 8927 5187 | Web: www.menzies.edu.au
ABN: 70 413 542 847

discovery for a healthy tomorrow

I. As the reviewer states, the decline in polyclonality and complexity of infection was consistent with transmission decline (described in the discussion in lines 258-272).

II. The increase in proportion of multiply observed multi-locus genotypes (moMLGs) was also consistent with declining transmission since this may reflect increasing inbreeding as a result of a shrinking population size (described in the text in lines 308-319).

III. Lastly, the observed increase in LD is also consistent with declining transmission since it may also reflect increased inbreeding as a result of a shrinking population - in the absence of large outbreaks. We have revised the text on lines 321-324 to highlight the consideration of outbreaks as follows:

"Since mixed-clone infections are a main contributor of cross-fertilisation events, LD is expected to correlate negatively with the proportion of polyclonal infections and thus, theoretically, should increase as the transmission intensity decreases in the absence of outbreaks. There was no evidence of large outbreaks of one or a few genetic strains in the *P. vivax* or *P. falciparum* populations in our study"

We have also included a neighbour-joining tree (Supplementary Figure 3), described on lines 212-213 supporting the statement that there was no evidence of large outbreaks of one or a few genetic strains.

No change in genetic diversity was observed during the study period. This is exactly what the study in reference 17 has shown along the Thai-Myanmar border. Interestingly, no change in genetic diversity of parasites (and no evidence of bottleneck) was found in another setting, Sri Lanka, with a much more drastic decrease in malaria transmission (doi: 10.1017/S0031182013002278, not cited in the text).

As stated by the reviewer, and in the text on lines 275-276 and 283-285, we did not find evidence of a significant decline in population-level diversity in *P. falciparum* or *P. vivax* – only a trend of declining diversity was observed in *P. falciparum*. We have revised the text on line 274 to clarify that "population genetic diversity has been proposed to correlate positively with endemicity" i.e. it is not always a robust correlate of transmission.

The reviewer is correct that Nkhoma and colleagues also failed to find evidence of a decline in population diversity *P. falciparum* over time in the Thai-Myanmar border region. We have added a reference to this study in the text on lines 277-278 as follows:

"Indeed, Nkhoma and colleagues also found population diversity to be limited as a measure of endemicity in their longitudinal survey of *P. falciparum* on the Thai-Myanmar border [17]".

We suspect that the trends in population genetic diversity observed in the referenced study in Sri Lanka may in part reflect imported cases. We had previously mentioned the potential contribution of imported cases to the local population diversity on lines 279-281 and 285. We have now also added a reference to the Sri Lanka study on lines 281-283 as follows:

PO Box 41096, Casuarina NT 0811, Australia
John Mathews Building (Bldg 58),
Royal Darwin Hospital Campus, Rocklands Dve, Casuarina NT 0810
Ph: 08 8922 8196 | Fax: 08 8927 5187 | Web: www.menzies.edu.au
ABN: 70 413 542 847

"A study of *P. vivax* population diversity in Sri Lanka reported increasing diversity despite declining transmission, potentially reflecting imported cases amongst other factors [19])."

I have previously mentioned that the study does not provide formal evidence for a population bottleneck in parasite populations. (I would guess that, even if properly tested, the results would still be negative.) However, even if the authors found evidence for bottleneck, this does not necessarily "confirm" that transmission has been interrupted or even decreased. A selective sweep induced by the widespread of a new drug, for example, might lead to genetic changes at the population level that are consistent with a bottleneck, even if transmission levels have not been greatly affected.

As mentioned above in response to the reviewer's earlier comment on LD, we have revised the text on lines 321-324 to highlight the consideration of outbreaks. In addition, we have included a neighbour-joining tree (Supplementary Figure 3), described on lines 212-213 to affirm that there was no evidence of large outbreaks of one or a few genetic strains.

Finally, there is throughout the paper a relatively loose use of the term "structure". If there is significant LD, of course malaria parasites are structured into lineages or subpopulations that do not recombine as much as expected for a randomly mating population. Therefore, stating that "there was no population structure among the P. vivax isolates (line 207) is simply incorrect. One can cautiously say that the Bayesian clustering algorithm implemented in STRUCTURE software was unable to detect population structure in the sample analysed. Whether STRUCTURE is the best strategy for detecting "structure" in populations at LD is debatable (see the STRUCTURE manual for a nice discussion on this topic).

As suggested by the reviewer, we have revised the text on lines 219-220 to clarify for other readers that our statement is based on the results of STRUCTURE analysis:

"The Bayesian clustering algorithm implemented in STRUCTURE software was unable to detect population substructure among the *P. vivax* isolates analysed (S3 Fig)."

The most likely cause of LD is the reduced proportion of multiple-clone infections documented during the study period, although it is biologically not true that "cross-fertilisation is only possible in mixed-clone infections" (as stated in line 280). Superinfection in the mosquito (due, e.g., to interrupted feeding) may also allow for cross-fertilisation.

We appreciate that superinfections may occur in the mosquito as the result of interrupted feeding, but we cannot conceive of why a phenomenon such as interrupted feeding would change so substantially over time as to amount to the significant changes in polyclonality that we observe in our study.

Nonetheless, we have clarified in the text on lines 258-261 that superinfections can indeed occur in the mosquito:

"Multiple clone infections can arise by superinfection in the mosquito (e.g. due to interrupted feeding) as well as superinfection in the patient following serial infected mosquito-bites. The risk of superinfection is likely to be greater in high transmission settings."

However, other factors may favour LD -- for example, if patient recruitment strategies have changed over time given the declining number of available patients, samples in more recent years may have been collected from a more (e.g., geographically) heterogeneous population where panmixia would be much less likely. These limitations must be recognised instead of overinterpreting LD results.

The patient recruitment strategy did not change at any point during the study period and hence the above factors are not likely to have contributed to the observed LD patterns. We have clarified this important point, which is a major strength of our study in the Methods on lines 397-399:

"The same sampling strategy was applied throughout the study period and ensures a homogenous patient catchment areas and demographics."

<u>Reviewer comments RE Summary and General Comments</u>

Overall, the paper is nicely written and describes important findings that are surely interesting to the broad audience of PLoS NTD. A better discussion of the data and, more specifically, their implications for malaria epidemiology, would render the paper even more interesting.

We thank the reviewer for their positive review of the manuscript. We believe that the suggested revisions have improved the overall study.
